# Vertical Distribution of Plant-Parasitic Nematodes in Sweet Potato

**DOI:** 10.2478/jofnem-2022-0025

**Published:** 2022-08-21

**Authors:** Bernard Kemboi, Hannah Karuri, Justine M. Nyaga, Aaron J. Kingsbury

**Affiliations:** 1 Department of Biological Sciences, University of Embu, Embu, Kenya; 2 Maine Maritime Academy, Castine, Maine 04420 United States

**Keywords:** ecology, gamma diversity, *Ipomoea batatas*, soil depth

## Abstract

Plant-parasitic nematodes (PPN) are harmful pests that have become a severe threat to crop production worldwide. Diversity of PPN at horizontal and spatial scales influence the effectiveness of control strategies. This study evaluated the vertical distribution of PPN genera at 0 cm to 30 cm and 30 cm to 60 cm in sweet potato fields in Central, Manyatta, and Nembure regions of Embu County, Kenya. A significant region × depth interaction was observed for *Tylenchus*. For all the other nematode genera, there were no significant variations in the abundance at 0 cm to 30 cm and 30 cm to 60 cm depths. However, *Helicotylenchus*, *Meloidogyne*, and *Scutellonema* occurred in greater numbers at both depths in all regions. Shannon and Simpson diversity indices were higher at 0 cm to 30 cm depth while Pielou’s evenness was similar at both depths in the three regions. Diversity partitioning of genus richness, Shannon, and Simpson diversities across all regions at 0 cm to 30 cm, indicated that β component contributed 61.9%, 35.6%, and 22.6% of γ diversity, respectively. Coinertia analysis indicated a significant covariation between nematode genera and soil properties. The results show that management of PPN in sweet potato fields should be targeted at soil depths that are not less than 60 cm.

Nematode distribution at different spatial scales is affected by several factors (Ou et al. 2005; Liu et al., 2019). Temperature, moisture, land use, soil properties, availability of nutrients, root architecture, and biomass are some of the variables that affect vertical distribution of nematodes (Fan-xiang et al., 2005; Liu et al., 2019). The level of crop damage by PPN is influenced by their distribution within the soil profile (Nguyen et al., 2020). For effective management of PPN, knowledge on their population densities at horizontal and vertical scales is imperative (Forge et al., 1998). Plant-parasitic nematodes damage crops through different modes of feeding within or outside the plant. They are broadly classified as ectoparasites, semi-endoparasites, migratory endoparasites, and sedentary endoparasites (Palomares-Rius et al., 2017). There are several PPN that are associated with sweet potato, including the economically damaging *Rotylenchulus reniformis* and *Meloidogyne* species that cause 5% to 10% yield losses (Hartemink et al., 2000; Loebenstein and Thottappilly, 2009; Jatala, 2019). In addition to these losses, *R. reniformis* causes cracking in storage roots, which affects quality and reduces their market value (Clark and Wright, 1983). In Kenya, root and tuber crops are the second most important staple food. Sweet potato contributes to food security and it also acts as a cash crop; it is a preferred crop for smallholder farmers due to its adaptability to different environmental conditions (MOALF, 2019). Plant-parasitic nematodes were identified as a major pest of sweet potato in Kenya and across East Africa (Echodu et al., 2019).

In cropping systems, the vertical distribution of PPN is variable at different depths, which influences their control. Ou et al. (2005) reported that PPN in paddy and maize fields decreased with depth, with the lowest abundance being at 80 cm to 100 cm. In a soybean field, *Paratrichodorus minor* and *Meloidogyne incognita* showed erratic vertical distribution and *Pratylenchus brachyurus* occurred in high numbers at 15 to 30 cm depth (McSorley and Dickson, 1990). Control of *Xiphinema index* using nematicides was ineffective due to the fact that the nematode inhabited deeper soil layers that were beyond the reach of the treatment (Villate et al. 2008). In a different study, control of *Meloidogyne xenoplax* and *Meloidogyne hapla* in *Vitis vinifera* was effective at 0 cm to 45 cm soil section compared to lower depths (Howland et al., 2014). Similarly, nematicide application at 0.5 m of the banana root system, where *Radopholus similis* and *Helicotylenchus multicinctus* were prevalent, was more effective (Kashaija et al., 2004).

In sweet potato, the use of resistant varieties (Kim and Yang, 2019), application of nematicides, plant extracts, green manure (Waisen et al., 2020), and organic amendments (Stirling, 2020, 2021) are among the methods used to control PPN. As demonstrated in some studies, the depth at which the PPN occurs is important for the efficacy of some of the control strategies. For instance, in potato and cabbage, there was an interaction between the depth at which *Belonolaimus longicaudatus* occurred and the effect of nematicide on the PPN. Population of *B. longicaudatus* was higher at 20 cm to 40 cm compared to 0 cm to 20 cm after nematicide application (Pérez et al., 2000). Application of the nematicide Ethoprop at 0 cm to 15 cm before planting sweet potato decreased the numbers of *Meloidogyne* and *Helicotylenchus* (Hall et al., 1988). Incorporation of green manure (sudangrass; Trudan 8) in soil columns with tomato plants reduced the abundance of *Meloidogyne chitwoodi* at the upper zone containing amendments, but not in the unamended bottom layers (Mojtahedi et al. 1993). At 0 cm to 60 cm depth, *X. index* population in a vineyard was reduced after application of chicken and sheep manure (Bello et al., 2004). The objective of this study was therefore to evaluate the diversity and distribution of PPN in sweet potato fields in Embu, Kenya at 0 cm to 30 cm and 30 cm to 60 cm. This information can be integrated in PPN management schemes in sweet potato cropping systems.

## Materials and Methods

### Study sites and sampling design

**Figure 1:**
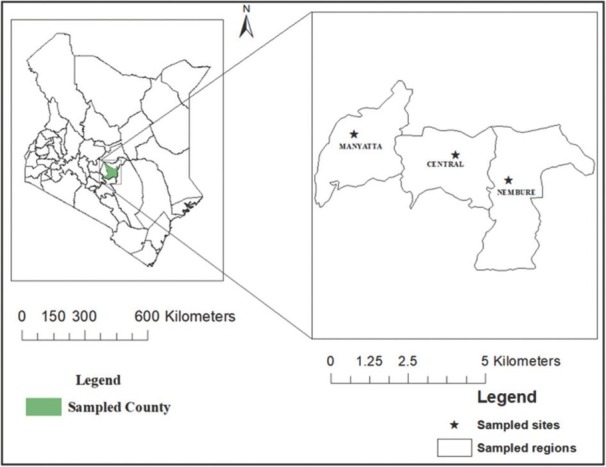
Soil sampling sites at Central, Manyatta, and Nembure regions in Embu County, Kenya.

Soil samples were collected from Central (0°31’26.9”S 37°26’52.3”E), Manyatta (0°28’36.1”S 37°26’18.0”E), and Nembure (0°28’28.7”S 37°26’46.2”E) regions of Embu County, Kenya (Fig. 1). Embu has two rainy seasons with annual rainfall and temperature of 1,000 mm to 2,000 mm and 12°C to 27°C, respectively. From each of the three regions, soil samples were collected from 15 fields, and in total 45 fields were sampled from the three sites. In every field, two composite soil samples were collected at 0 cm to 30 cm and 30 cm to 60 cm depths following the protocol described by Wiesel et al. (2015). Sweet potato was at approximately 3 months after planting and the fields had not received agrochemical inputs or pest management.

### Nematode identification

Modified Baermann technique was used to extract nematodes from triplicate 250 g of soil for 48 hr (Hooper, 1986). The nematodes were fixed using a hot fixative (Hooper, 1970) before identification using a microscope. Morphological keys (Bongers, 1988; Mai and Mullin, 1996) were used in identification of the nematodes up to genus level. Soil samples were taken to the Kenya Agriculture and Livestock Research Organization, National Agricultural Research Laboratories for analysis of soil properties.

### Data analysis

Two-way analysis of variance (ANOVA) was performed to determine the effect of region and depth on the abundance of nematode genera and diversity indices in Central, Manyatta, and Nembure regions. Means that were significantly different were separated using Tukey’s HSD test. For significant region × depth interactions, simple main effects analysis was conducted. Pielou’s evenness, genus richness, Shannon–Weaver, and Simpson diversity indices were determined using vegan library in R software. The same library was used to conduct additive diversity partitioning of nematode γ diversity using *adipart* function. Association between depth and nematode genera was examined by computing Pearson Phi coefficient (De Cáceres and Legendre, 2009) using *multipatt* function in indicspecies package of R software. Spatial distribution of nematode genera at 0 cm to 30 cm and 30 cm to 60 cm in the three regions was assessed using nonmetric multidimensional scaling (NMDS) based on the Jaccard index (Clarke and Ainsworth, 1993). Differences in nematode genera at the two depths were analyzed using permutational multivariate analysis of variance (PERMANOVA) and permutational multivariate analysis of dispersion (PERMDISP) (Anderson, 2001, 2006). The most influential nematode genera were determined using similarity percentage analysis. The relationships between soil properties and nematode communities were determined using coinertia analysis (CoIA) (Dolédec and Chessel, 1994, Dray et al., 2003) and correlation was given by the RV coefficient. Before CoIA, nematode abundance and soil properties were analyzed using principal component analysis. The significance of the correlation was determined by Monte Carlo permutations (999). Library ade4 of R software was used by applying the functions *coinertia* and *randtest* (Dray and Dufour, 2007).

## Results

**Figure 2:**
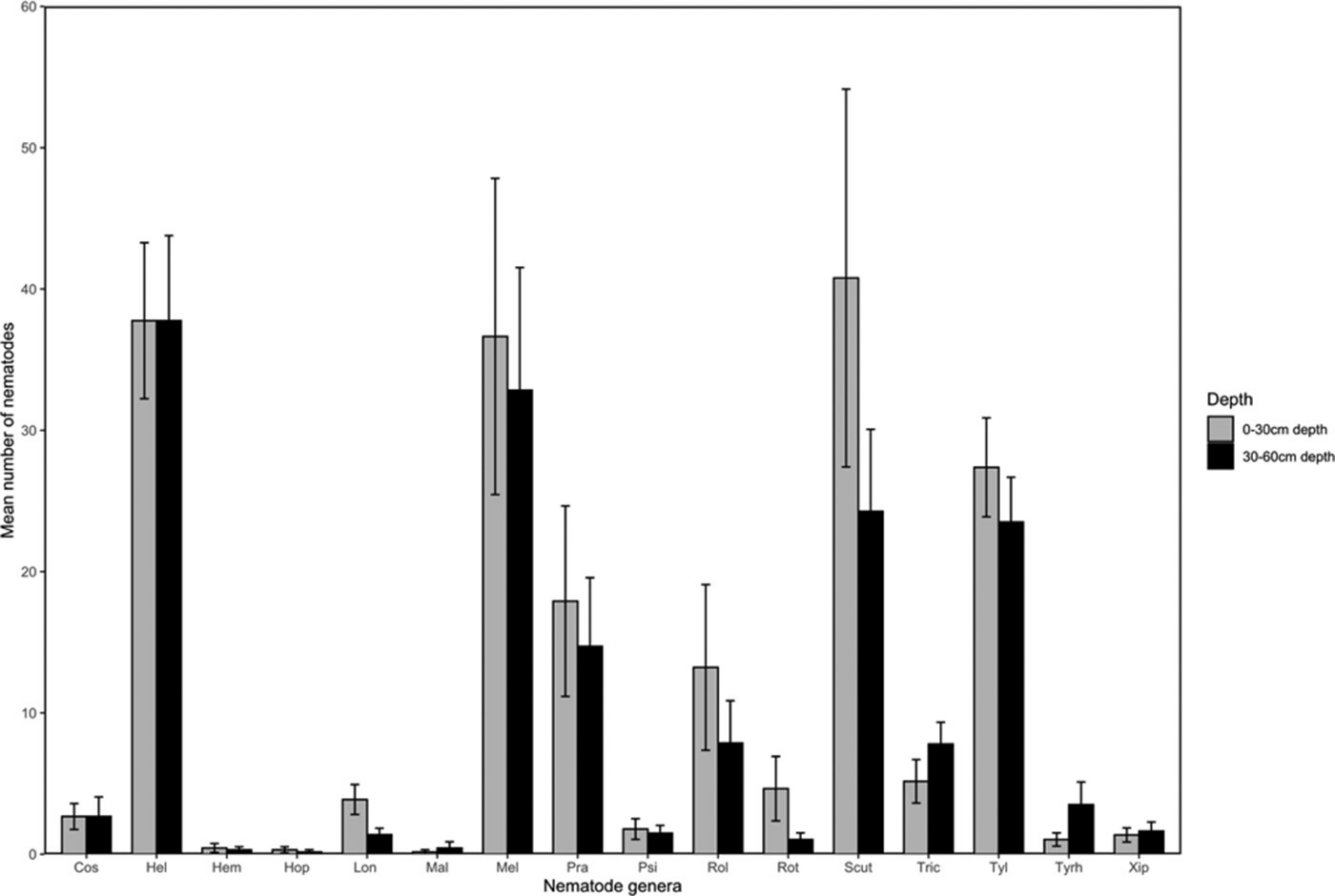
Abundance (mean ± standard error) of nematode genera in 250 g of soil from sweet potato fields in Central, Manyatta, and Nembure regions of Embu County, Kenya at 0 cm to 30 cm and 30 cm to 60 cm depths. Cos: *Coslenchus*; Hel: *Helicotylenchus*; Hem: *Hemicycliophora*; Hop: *Hoplolaimus*; Lon: *Longidorus*; Mal: *Malenchus*; Mel: *Meloidogyne*; Pra: *Pratylenchus*; Psi: *Psilenchus*; Rol: *Rotylenchulus*; Rot: *Rotylenchus*; Scut: *Scutellonema*; Tric: *Trichodorus*; Tyrh: *Tylenchorhynchus*; Tyl: *Tylenchus*; Xip: *Xiphinema*.

**Figure 3:**
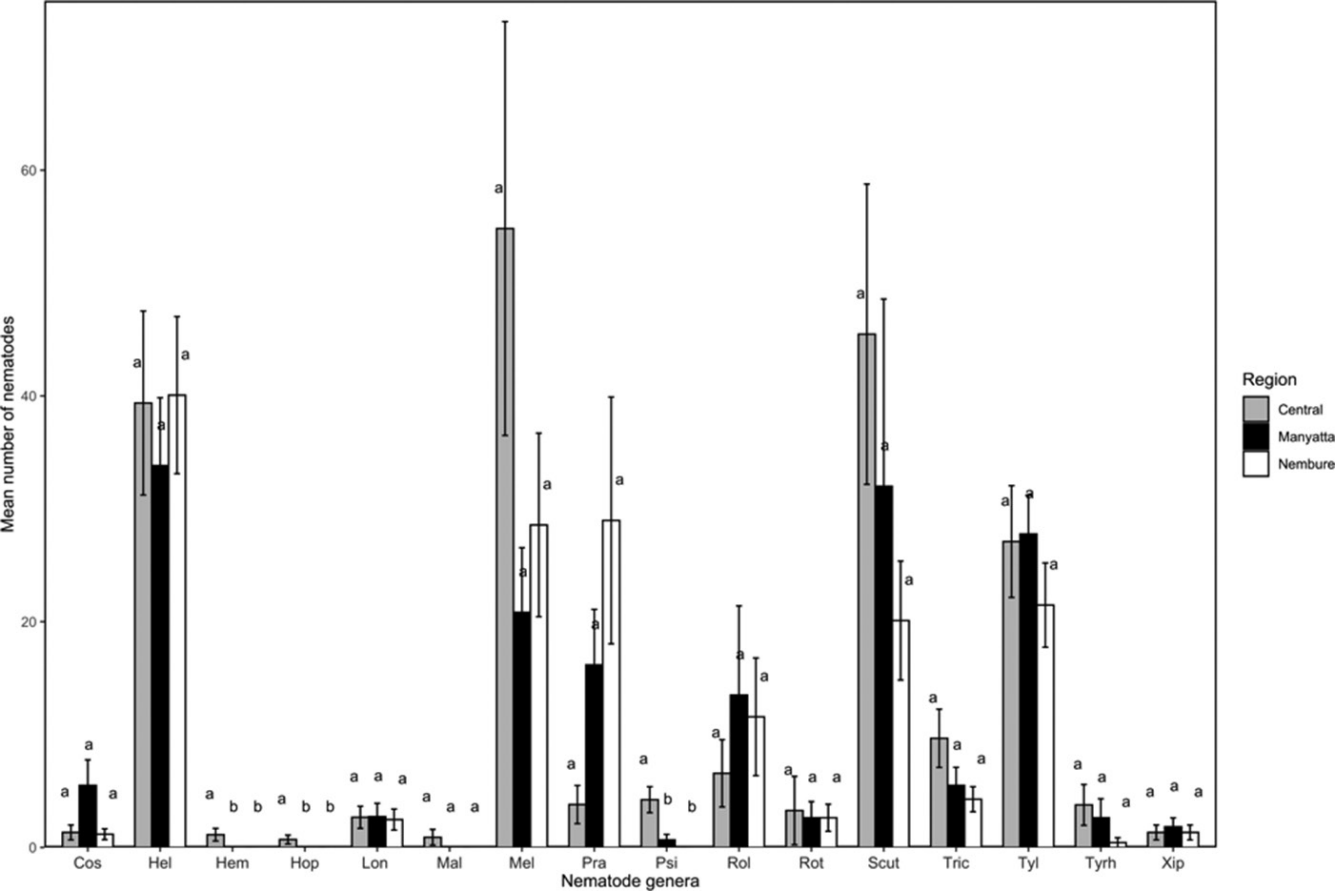
Abundance (mean ± standard error) of nematode genera in 250 g of soil from sweet potato fields in Central, Nembure, and Manyatta regions of Embu County, Kenya. Cos: *Coslenchus*; Hel: *Helicotylenchus*; Hem: *Hemicycliophora*; Hop: *Hoplolaimus*; Lon: *Longidorus*; Mal: *Malenchus*; Mel: *Meloidogyne*; Pra: *Pratylenchus*; Psi: *Psilenchus*; Rol: *Rotylenchulus*; Rot: *Rotylenchus*; Scut: *Scutellonema*; Tric: *Trichodorus*; Tyrh: *Tylenchorhynchus*; Tyl: *Tylenchus*; Xip: *Xiphinema*.

Sixteen nematode genera were observed across Central (16), Manyatta (13), and Nembure (12) regions of Embu. Two-way ANOVA indicated a significant variation in abundance of *Psilenchus* (*P* < 0.0001), *Hemicycliophora* (*P* = 0.01), and *Hoplolaimus* (*P* = 0.01) across the regions. A significant region × depth interaction was observed for *Tylenchus* (*P* = 0.01) (Table 1; Figs. 2 and 3). From the simple main effects analysis, it could be ascertained that there were lower populations of *Tylenchus* in Central region at 30 cm to 60 cm depth compared to Manyatta and Nembure. At 0 cm to 30 cm depth, the populations were higher in Central than in Nembure. For all the other nematode genera, there were no significant variations in the abundance at 0 cm to 30 cm and 30 cm to 60 cm depths. However, *Helicotylenchus*, *Meloidogyne*, and *Scutellonema* occurred in relatively high numbers at both depths in all the regions. Pearson’s phi coefficient of association showed that *Scutellonema* was significantly associated with 0 cm to 30 cm depth (r = 0.262; *P =* 0.017). Region and depth significantly influenced Shannon–Weaver (*P* = 0.04) and Simpson (*P* = 0.05) diversities (Table 1). Across all regions, Shannon–Weaver and Simpson diversity indices were higher at 0 cm to 30 cm depth (Table 2) while Pielou’s evenness was similar at both depths (J = 0.81 ± 0.02, 0–30 cm; J = 0.79 ± 0.02, 30–60 cm).

**Table 1:** Two-way analysis of variance (P values) of region, depth, and their interaction on nematode genera, Pielou’s evenness, Shannon–Weaver, and Simpson diversity indices in sweet potato fields in Central, Manyatta, and Nembure regions of Embu County, Kenya.

	Region	Depth	Region × depth
**Nematode genera**			
*Coslenchus*	0.11	0.64	0.72
*Helicotylenchus*	0.97	0.97	0.93
*Longidorus*	0.98	0.08	0.14
*Meloidogyne*	0.4	0.35	0.71
*Pratylenchus*	0.1	0.91	0.81
*Rotylenchulus*	0.83	0.68	0.83
*Rotylenchus*	0.65	0.36	0.96
*Scutellonema*	0.08	0.81	0.75
*Trichodorus*	0.49	0.17	0.3
*Tylenchorhynchus*	0.06	0.33	0.83
*Tylenchus*	0.15	0.32	0.01*
*Xiphinema*	0.78	0.9	0.07
*Psilenchus*	<0.0001***	0.93	1
*Hemicycliophora*	0.01*	0.9	0.98
*Hoplolaimus*	0.05*	0.56	0.71
*Malenchus*	0.15	0.79	0.93
**Diversity indices**			
Shannon–Weaver diversity	0.04*	0.03*	0.53
Simpson diversity	0.08	0.05*	0.84
Pielou’s evenness	0.89	0.41	0.24

Asterisks represent level of significance: ***P < 0.0001, *P < 0.05.

**Table 2:** Simpson and Shannon–Weaver diversity indices of nematode communities in sweet potato fields in Central, Manyatta, and Nembure regions in Embu County, Kenya at 0 cm to 30 cm and 30 cm to 60 cm depths.

Region	Shannon–Weaver diversity		Simpson diversity	
	Mean	SE	Mean	SE
Central	1.29^a,b^	0.06	0.64^a^	0.02
Manyatta	1.36^a^	0.06	0.67^a^	0.02
Nembure	1.12^b^	0.08	0.58^a^	0.04
**Depth**				
0–30 cm	1.38^a^	0.05	0.68^a^	0.02
30–60 cm	1.15^b^	0.06	0.59^b^	0.03

Means with the same letter along a column are not significantly different. SE, standard error of mean.

**Figure 4:**
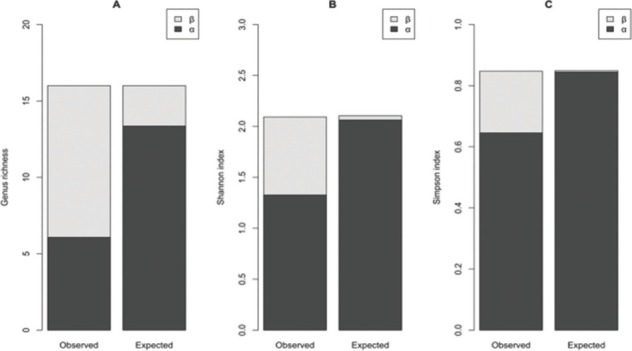
Additive γ diversity partitioning (into α and β components) of (A) nematode genus richness, (B) Shannon–Weaver diversity, and (C) Simpson diversity in sweet potato fields in Central, Manyatta, and Nembure regions in Embu County, Kenya at 0 cm to 30 cm and 30 cm to 60 cm depths.

**Figure 5:**
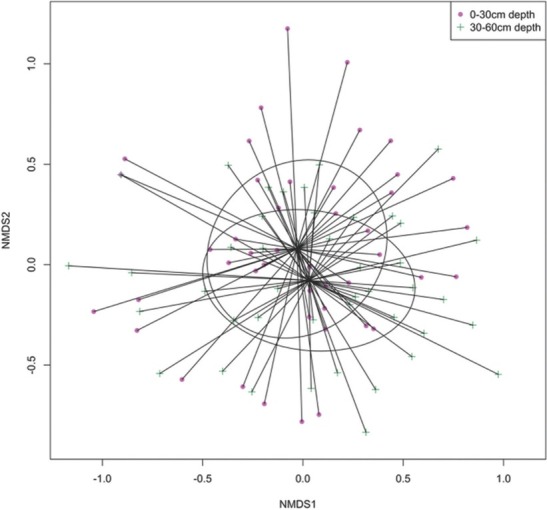
NMDS ordination of nematode communities in sweet potato fields in Central, Manyatta, and Nembure regions in Embu County, Kenya at 0 cm to 30 cm and 30 cm to 60 cm depths (NMDS Stress = 0.2). NMDS, nonmetric multidimensional scaling.

**Figure 6:**
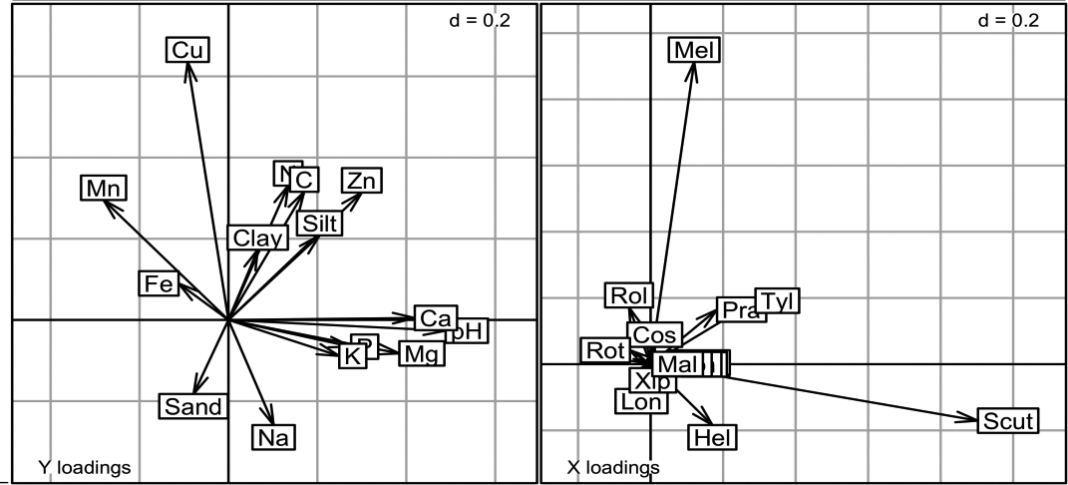
Coinertia analysis of nematode genera and soil properties at 0 cm to 30 cm depth in Central, Manyatta, and Nembure regions in Embu County, Kenya (RV = 0.13, *P* = 0.05, Monte Carlo permutation test).

Diversity partitioning of genus richness, Shannon–Weaver, and Simpson diversities across the three regions, at 0 cm to 30 cm and 30 cm to 60 cm depths, indicated that β component contributed 61.9%, 35.6%, and 22.6% of γ diversity, respectively (Fig. 4). In the NMDS analysis, nematode community structure was not clearly differentiated at the two depths in all the regions and the average dissimilarity was 67.4% (Fig. 5; PERMANOVA, R^2^ = 0.005, F = 0.44, *P* = 0.99; PERMDISP, F = 0.015, *P* = 0.90). The most influential genera that contributed to the NMDS structure were *Helicotylenchus*, *Meloidogyne, Scutellonema*, *Tylenchus*, and *Pratylenchus*. Across the three regions, there were significant differences in N, C, Mn, Zn, Na, and silt at 0 cm to 30 cm depth (Table 3). According to CoIA, there was significant covariation (RV = 0.13, *P* = 0.05; Monte Carlo test) between nematode genera and soil properties. The first and second axes accounted for 60.4% and 27.6% of the total variance, respectively. There were positive correlations between pH, Ca, Mg, and *Scutellonema*. Population density of *Tylenchus* was positively linked to N, C, silt, and Zn while *Meloidogyne* was associated with high Cu levels (Fig. 6).

**Table 3:** Soil properties in sweet potato fields at 0–30 cm depth in Central, Manyatta, and Nembure regions in Embu County, Kenya.

Soil property	Central		Manyatta		Nembure		P value
	Mean	SE	Mean	SE	Mean	SE	
pH	4.89	0.23	4.70	0.16	4.72	0.14	0.74
Total Nitrogen%	0.36	0.01	0.38	0.02	0.29	0.03	0.02*
Total Organic Carbon%	4.04	0.14	4.25	0.20	3.36	0.31	0.02*
Phosphorus ppm	25.31	4.43	17.33	2.09	14.86	2.76	0.08
Potassium meq%	0.92	0.16	1.10	0.12	0.84	0.14	0.42
Calcium meq%	2.56	0.69	2.55	0.65	1.81	0.62	0.67
Magnesium meq%	3.39	0.34	3.29	0.33	3.29	0.28	0.97
Manganese meq%	0.79	0.04	0.85	0.05	0.66	0.05	0.02*
Copper ppm	4.83	2.09	3.55	1.34	1.26	0.30	0.26
Iron ppm	27.25	4.15	33.38	7.11	26.68	5.00	0.64
Zinc ppm	34.12	3.49	23.01	2.54	16.18	2.47	<0.0001***
Sodium meq%	0.58	0.03	0.44	0.03	0.46	0.04	0.02*
Sand (%)	10.00	3.89	15.87	5.50	13.71	6.24	0.72
Clay (%)	77.63	3.73	72.13	5.28	75.29	6.15	0.74
Silt (%)	12.38	0.33	12.00	0.34	11.00	0.35	0.02*

Asterisks represent level of significance: *P < 0.05, ***P < 0.0001. SE, standard error of mean.

## Discussion

Vertical distribution of PPN determines the effectiveness of control strategies (Ingham et al., 2000). *Helicotylenchus*, *Meloidogyne*, and *Scutellonema* occurred in relatively high numbers at 0 cm to 30 cm and 30 cm to 60 cm depths in all the regions but the abundance was not significantly different. However, *Scutellonema* showed a specific association with 0 cm to 30 cm depth. Similarities in nematode genera at the two depths were also depicted in the NMDS analysis. These nematodes have been previously reported in association with sweet potato in Kenya (Njuguna and Bridge, 1998). Lack of significant differences at the two soil depths may be due to the influence of crop growth stage on PPN population dynamics whereby similar numbers of nematodes may be present either in the soil or within the plant at a particular time (Sharma et al., 1992). Root density (Robinson et al., 2005) and architecture also influence the vertical distribution of PPN (Pudasaini et al., 2006, Wesemael and Moens, 2008). The population density of PPN within plant roots at 0 cm to 30 cm and 30 cm to 60 cm depths was not tested in the current study, and this avenue of study warrants further investigation. Based on soil conditions, the sweet potato root system may occur up to 2-m deep (Woolfe, 1992), which may explain the uniform distribution of most of the PPN genera within the soil profile (Fan-xiang et al., 2005). At depths below 40 cm, Rodriguez-Kabana and Robertson (1987) also observed constant distribution of *Meloidogyne arenaria*, which they attributed to light soil texture.

Contrary to our observations, *Scutellonema* and *Helicotylenchus* have been reported to decrease with depth in most crops. In a groundnut field, >95% of *Scutellonema clathricaudatum* were found at 0 cm to 30 cm (Sharma et al., 1992) while a higher population of *Helicotylenchus* was observed at 20 cm to 30 cm in a fallow field (Fan-xiang et al., 2005). Similarly, Araya and De Waele (2011) observed a decline in the number of *Helicotylenchus* at higher soil depths in a banana field. In the current study, population densities of *Tylenchus* were variable at both depths in the three regions, which is similar to observations reported elsewhere in the literature (Fan-xiang et al., 2005, Siddiqui, 2007). *Meloidogyne* species are considered the most economically damaging PPN in sweet potato and they cause 10% to 20% of yield losses (Koenning et al., 1999; Okechalu and Wonang, 2015). The losses resulting from RKN infestation may be variable depending on the nematode species (Overstreet, 2009). Root-knot nematodes also cause necrosis and predispose the storage roots to cracking, which reduces their market value (Lawrence et al. 1986). High population density of *Helicotylenchus* reduces the weight of tubers, roots, and shoot biomass (Lopez et al., 1981). Although *Scutellonema* (Njuguna and Bridge, 1998; Coyne et al., 2003) and *Tylenchus* (Haougui et al., 2011) are associated with sweet potato, there are limited studies on their effects on yield.

Effective control of PPN that were observed in this study will require that information on their vertical distribution is considered. This was demonstrated by Ingham et al. (2000), where *Meloidogyne chitwoodi* was not controlled by metam sodium, ethoprop, and oxamyl at 0 cm to 30 cm due to migration of the nematode from 120-cm depth to the upper soil layers. In a different study, solarization at 40-cm soil depth was more effective in controlling *M. incognita* eggs (Nico et al., 2003). Rodriguez-Kabana and Robertson (1987) also suggested that for control of *M. arenaria*, placement of fumigant nematicides that move upwards should be such that they cover the depth where the nematodes are most prevalent. Low-cost PPN management techniques in sweet potato would be preferred by smallholder farmers in Kenya. The optimal depth at which the treatments would have a higher efficacy against the PPN should be tested on a case-by-case basis.

Shannon and Simpson diversities were higher at 0 cm to 30 cm across Central, Manyatta, and Nembure regions with the a component having a higher contribution to γ diversity. Zheng et al. (2012) also observed a decrease in these indices at higher soil depths, which was corroborated by Zhong et al. (2015) in different cropping systems. Processes that shape PPN populations such as niche differentiation, competition, reproduction, r-k strategies, and feeding methods affect their diversity and influence their response to control agents (Pérez et al., 2000; Pudasaini et al., 2006). Effect of occurrence of multiple PPN on crop damage was demonstrated in sweet potato, whereby competition between *M. incognita* and *R. reniformis* during concomitant infection resulted in the dominance of one species (Thomas and Clark, 1983). Co-occurrence of multiple PPN in soil influences the damage that they cause in crops (Thomas and Clark, 1983) and hence the need to consider this aspect when choosing nematode control strategies.

Regional differences that were observed in diversity indices and PPN abundance may be explained by several factors including soil properties (Hoogen et al., 2019; Li et al., 2020). Soil properties are an important factor in shaping distribution of nematode communities at different spatial levels (Nguyen et al., 2020). In the present study, *Scutellonema* was positively correlated with pH, Ca, and Mg, while Tylenchus was positively correlated with N, C, silt, and Zn. The abundance of *Meloidogyne* was associated with high Cu levels, as previously observed by Krif et al. (2020). However, according to Noe (1985), *Meloidogyne incognita* prefers soil with low Cu concentration due to its toxicity. Association of *Scutellonema* with high pH and Mg was reported in Western Kenya (Kandji et al., 2001). Aït Hamza et al. (2018) reported a positive correlation between clay and *Tylenchus*, unlike what was observed in the present study. Texture is important in the movement of PPN in the soil profile (Wesemael and Moens, 2008), whereby motility is faster where the silt/clay content is low (Prot and Gundy, 1981).

Apart from soil properties, other factors such as season, crop type (Nguyen et al., 2020), tillage (Lenz and Eisenbeis, 2000), soil aggregates, interactions with other organisms (Liu et al., 2019), and density-and time-dependent factors (Eisenback, 1985), which were not considered in this study, may also contribute to differences in diversity of PPN at different depths. From our results, we find that PPN distributions for most genera were similar across the 0 cm to 30 cm and 30 cm to 60 cm depths. There is a possibility that the abundance of PPN observed in this study was higher at depths >60 cm, an inference that warrants further investigation before a definitive conclusion can be reached. Control of PPN in sweet potato using treatments that are applied at depths below 60 cm may not effectively eliminate a great proportion of the nematodes. Since the population dynamics of PPN in sweet potato fields may be affected by the aforementioned factors, bespoke integrated nematode management schemes in sweet potato cropping systems need to be designed.
